# Polygenic risk for major depression, attention deficit hyperactivity disorder, neuroticism, and schizophrenia are correlated with experience of intimate partner violence

**DOI:** 10.1038/s41398-024-02814-1

**Published:** 2024-02-26

**Authors:** Andrew Ratanatharathorn, Luwei Quan, Karestan C. Koenen, Lori B. Chibnik, Marc G. Weisskopf, Natalie Slopen, Andrea L. Roberts

**Affiliations:** 1 Department of Epidemiology, Columbia University Mailman School of Public Health, New York, NY USA; 2 Department of Epidemiology, Harvard T.H. Chan School of Public Health, Boston, MA USA; 3 Department of Environmental Health, Harvard T.H. Chan School of Public Health, Boston, MA USA; 4 Department of Social and Behavioral Sciences, Harvard T.H. Chan School of Public Health, Boston, MA USA; 5 Broad Institute of MIT and Harvard, Cambridge, MA USA; 6 Department of Psychiatry, Massachusetts General Hospital, Boston, MA USA; 7 Department of Neurology, Massachusetts General Hospital, Boston, MA USA

**Keywords:** Genetics, Human behaviour

## Abstract

Research has suggested that mental illness may be a risk factor for, as well as a sequela of, experiencing intimate partner violence (IPV). The association between IPV and mental illness may also be due in part to gene-environment correlations. Using polygenic risk scores for six psychiatric disorders - attention-deficit hyperactivity disorder (ADHD), autism spectrum disorder (ASD), bipolar disorder (BPD), major depressive disorder (MDD), neuroticism, and schizophrenia—and a combined measure of overall genetic risk for mental illness, we tested whether women’s genetic risk for mental illness was associated with the experience of three types of intimate partner violence. In this cohort of women of European ancestry (*N* = 11,095), participants in the highest quintile of genetic risk for ADHD (OR range: 1.38–1.49), MDD (OR range: 1.28–1.43), neuroticism (OR range: (1.18–1.25), schizophrenia (OR range: 1.30–1.34), and overall genetic risk (OR range: 1.30–1.41) were at higher risk for experiencing more severe emotional and physical abuse, and, except schizophrenia, more severe sexual abuse, as well as more types of abuse and chronic abuse. In addition, participants in the highest quintile of genetic risk for neuroticism (OR = 1.43 95% CI: 1.18, 1.72), schizophrenia (OR = 1.33 95% CI: 1.10, 1.62), and the overall genetic risk (OR = 1.40 95% CI: 1.15, 1.71) were at higher risk for experiencing intimate partner intimidation and control. Participants in the highest quintile of genetic risk for ADHD, ASD, MDD, schizophrenia, and overall genetic risk, compared to the lowest quintile, were at increased risk for experiencing harassment from a partner (OR range: 1.22–1.92). No associations were found between genetic risk for BPD with IPV. A better understanding of the salience of the multiple possible pathways linking genetic risk for mental illness with risk for IPV may aid in preventing IPV victimization or re-victimization.

In the United States, an estimated 37% of women will experience sexual violence, physical violence, or stalking by an intimate partner in their lifetimes [1]. Following an experience of intimate partner violence (IPV), women are more likely to also experience physical injury [2], job loss [3], and poorer mental health [1]. Poorer mental health includes mental illness, which has been found to precede [4–7] as well as follow [6, 7] experiences of IPV, indicating that mental illness may be both a risk factor for and sequela of IPV. It is also possible that IPV and mental illness co-occur in part due to heritable genetic risk for mental illness [8]. Better understanding of the salience of the multiple possible pathways linking mental illness with risk for IPV may aid in treating IPV victimization or preventing re-victimization [8].

Co-occurrence between the environmental experience of IPV and genetic risk for mental illness is an example of gene-environment correlation (rGE), which could arise through at least two pathways (Fig. 1) [8]. In the passive rGE pathway, parental genetic risk for mental illness increases the likelihood of an adverse childhood environment for offspring, such as experiencing childhood abuse [9–11], low socioeconomic status (SES) [12], and parental divorce [9, 13–15], which could then increase risk of offspring experiencing IPV. As offspring also inherit genetic risk for mental illness [16], a correlation is induced between offspring’s experience of IPV and mental illness. In the evocative rGE pathway, offspring genetic risk for mental illness may increase the likelihood of experiencing risk factors for IPV victimization, such as social isolation [7, 17, 18] or insecure attachment [19, 20], or traits, such as low self-esteem [21, 22], deficits in emotional dysregulation [23, 24], or reduced ability to interpret facial expressions [25–27], that are associated with increased risk of IPV.

**Fig. 1:**
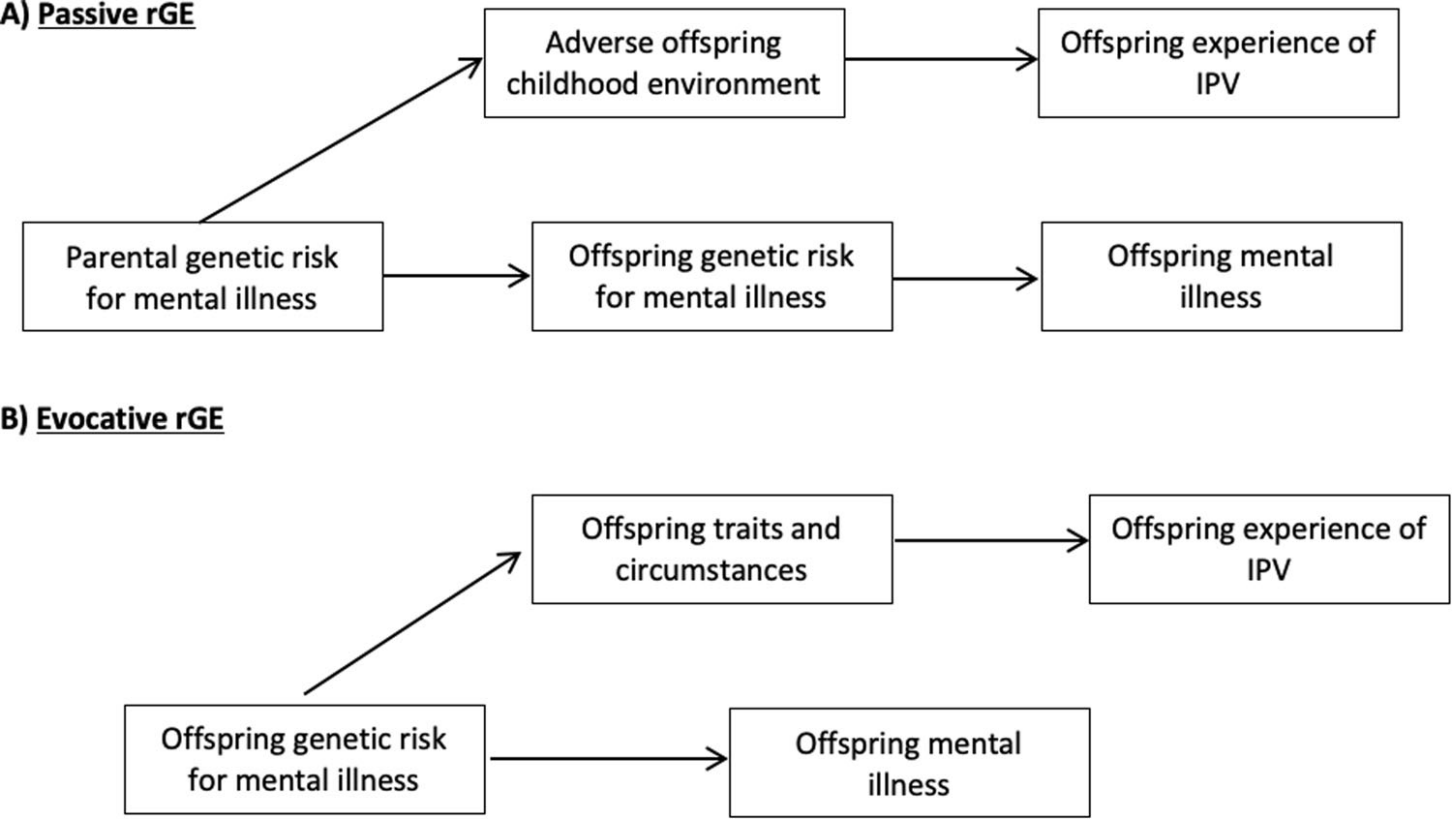
Potential pathways by which genetic risk for mental illness may be associated with intimate partner violence.

Whether rGE exists between genetic risk for mental illness and experience of IPV may be informative to clinicians treating people with experience of IPV. For example, the experience of IPV may indicate a genetic liability for depression. Over time, the clinical utility of genotyping in determining the best pharmacological and psychotherapy treatment may improve, along with our understanding of the role of gene-by-environment interactions. If so, genotyping patients with depression and history of IPV may result in more personalized treatment [28, 29]. However, to date, only a single twin study has examined rGE between depression and IPV finding modest evidence for rGE [30], but no study has tested whether genetic risk for a range of mental illnesses and genetic traits are correlated with the experience of IPV using genetic data. To address this, we estimated individual-level genetic risk for mental illness using polygenic risk scores (PRS) from publicly available summary statistics of genome-wide association studies of attention deficit hyperactivity disorder (ADHD) [31], autism spectrum disorders (ASD) [32], bipolar disorder (BPD) [33], major depressive disorder (MDD) [34], neuroticism [35], and schizophrenia (SCZ) [36] in a large cohort of US women, the Nurses’ Health Study II (NHS2), to examine whether genetic risk for mental illness was associated with experience of IPV. We additionally examined whether PRS was associated with experiencing multiple types of victimization (e.g., emotional, physical, sexual), chronic victimization, and harassment or stalking.

## Materials and methods

The NHS2 is an ongoing cohort of 116,430 female nurses recruited in 1989 and assessed every two years. Participants were ages 24–44 years at baseline. Blood samples were collected from 29,611 participants between 1996 and 1999, as previously described [37]. Genome-wide data was available for 13,313 women based on three genotyping platforms: (1) Illumina Human Hap Array (*N* = 781), (2) Illumina OncoArray (*N* = 2722), and 3) Illumina HumanCore Exome Chip (Batch 1 *N* = 3276; Batch 2 *N* = 4568). Of these 13,313 participants, 11 701 (87.9%) completed questions assessing intimate partner violence. Following a standard quality control pipeline (call rate >0.90), participant genotype data were imputed using 1000 Genomes phase 3 reference data [38]. We included only participants of European ancestry (*N* = 11,344) as <2% of participants reported non-European ancestries and previous findings that PRS for mental illness developed from GWAS of European ancestry participants do not perform well for non-European ancestries due to differences in linkage disequilibrium patterns and frequency of alleles [39]. Informed consent was received from all participants. The study protocol was approved by the Institutional Review Boards of the Brigham and Women’s Hospital and the Harvard T.H. Chan School of Public Health (protocol number: 2012P002031).

### Polygenic risk scores

PRS for ADHD [31], ASD [32], BPD [33], MDD [34], neuroticism [35], and schizophrenia [36] were calculated using the summary statistics from the largest published GWAS, with *p* value thresholds, clumping parameters, minor allele frequencies, and imputation score cutoffs based on those found to explain maximum variance based on Nagelkerke’s *R*^2^ from each analysis (see Supplementary Table 1) [40, 41]. Participant’s PRS for each mental illness was calculated by taking the weighted sum of risk alleles, with each allele weighted by the log odds ratio reported in published GWAS summary statistics using PRSice-2 [31–36, 42, 43]. PRS were then standardized using *z* score transformations. An overall PRS for mental illness for each participant was created by summing the six standardized PRS and then standardizing the result. Quintiles of genetic risk for each mental illness and the overall PRS were then calculated for each participant.

### Intimate partner violence

In 2001, three sets of questions were used to assess experiences of IPV. First, participants were asked about lifetime occurrence of three types of abuse by their spouse or significant other: emotional (“ever been emotionally abused”); physical (“ever been hit, slapped, kicked, or otherwise physically hurt); and sexual (“ever forced to have sexual activities”). For each type, participants could respond “no, this never happened”, “yes, this happened once”, or “yes this happened more than once.” They were then asked to indicate the calendar years in which they experienced any of these types of abuse (1962–2001). To estimate severity of IPV, we calculated number of types of abuse as a count of types a respondent reported experiencing more than once (range: 0–3). We calculated abuse chronicity as the number of years in which they experienced abuse (0, 1, 2–3, or 4+ years).

Second, the experience of intimate partner intimidation and control was assessed with the 10-item Relationship Assessment Tool (RAT), which queried the degree to which women felt fearful, ashamed, disempowered, and controlled by their significant other [44–46] (e.g., “my partner can scare me without laying a hand on me”; “my partner makes me feel I have no control over my life…”), with responses ranging from 1: “strongly agree” to 6: “strongly disagree”. Scores on the RAT range from 10 to 60, with 10 indicating no feelings of intimidation and control and 60 indicating severe intimidation and control. Based on prior research, the scale was dichotomized at ≥20 to indicate the experience of intimate partner intimidation and control [46].

Third, eight questions based on the National Violence Against Women Survey assessed experience of harassment or stalking, including having someone spying or standing outside their home, school, or workplace; receiving unwanted letters, phone calls, or items; having property vandalized; or having to file a restraining order [47]. Participants were asked whether the perpetrator was a spouse or significant other, ex-spouse or ex-significant other, or other person. We defined harassment as having experienced any of these circumstances and created separate variables for history of harassment perpetrated by: 1) partners (spouses/significant others); 2) ex-partners (ex-spouses/ex-significant others); and 3) other persons, each coded any/none.

### Covariates

To account for residual population stratification—systematic differences in allele frequencies across ancestries that can lead to spurious results—we included 10 principal components derived from the genetic data as covariates [48].

### Statistical analyses

Pearson correlations between each pair of PRS were calculated to examine the relationships between PRS. To ascertain whether genetic risk for mental illness was associated with emotional, physical, or sexual IPV, we fit separate ordinal logistic regressions with each type of IPV (emotional, physical, sexual) as the dependent variable coded as none, once, or more than once, and quintile of genetic risk as the independent variable. To estimate the association of PRS with number of types and chronicity of abuse, we fit separate ordinal logistic models to estimate odds ratios (ORs) of experiencing more severe abuse or more chronic abuse in association with a quintile of polygenic risk for each mental illness. The validity of the proportional odds assumption was assessed using Brant tests [49] and a likelihood test comparing an ordinal logistic model and multinomial (unconstrained) logistic model. Next, we fit a logistic regression to estimate the association between polygenic risk and lifetime experience of intimate partner intimidation and control, coded any/none. Finally, we estimated odds of lifetime experience of harassment perpetrated by: (1) partners; (2) ex-partners; and (3) others, with separate logistic regressions for each. To test whether linear increases in PRS for each trait were associated with an outcome, we refit models coding PRS as a continuous variable rather than in quintiles. All models adjusted for genomic assay and top 10 principal components of genetic ancestry. To assess the independent effect of each PRS, models were refit including the PRS of ADHD, ASD, BPD, MDD, neuroticism, and schizophrenia. Analyses were performed using R version 4.02 [50].

## Results

One-quarter of participants reported experiencing physical IPV (once: 13.2%, more than once: 11.1%), 41.5% reported emotional IPV (once: 8.4%, more than once: 33.1%) and 12.3% reported sexual IPV (once: 5.7%, more than once 6.6%, Table 1). Overall, 21.4% of participants reported experiencing at least one type of abuse more than once, and 3.4% reported experiencing all three types of abuse more than once. 13.5% of participants reported experiencing intimate partner intimidation and control. Regarding experiences of harassment, women experienced these perpetrated by a partner least often (9.2%), followed by an ex-partner (13.3%), and then by someone else (15.7%). In our analysis of correlations among PRS, we found, first, that ADHD and BPD (*r* = 0.63), BPD and schizophrenia (*r* = 0.48), and ADHD and schizophrenia (*r* = 0.38) were positively correlated with each other and negatively correlated with neuroticism (*r* range: −0.17 to −0.29) and MDD (r range: −0.04 to −0.15). Second, we found that neuroticism and MDD were correlated with each other (*r* = 0.26). ASD was not correlated with any other PRS (see Supplementary Fig. S1).

**Table 1:** Descriptive statistics for intimate partner violence, intimate partner intimidation and control, harassment, and demographic variables of the Nurses Health Study II participants.

Variable	Parameter
*N*	11,344
Age, mean (SD)	46.6 (4.4)
Experienced emotional abuse, *N* (%)	
No	6490 (58.5%)
Once	937 (8.4%)
More than once	3668 (33.1%)
Experienced physical abuse, *N* (%)	
No	8415 (75.7%)
Once	1469 (13.2%)
More than once	1237 (11.1%)
Experienced sexual abuse, *N* (%)	
No	9742 (87.7%)
Once	639 (5.7%)
More than once	733 (6.6%)
Number of types of abuse experienced more than once, *N* (%)	
0	7272 (65.6%)
1	2377 (21.4%)
2	1061 (9.6%)
3	375 (3.4%)
Number of years abuse experienced, *N* (%)	
0 years	6348 (56.0%)
1 year	1321 (11.6%)
2–3 year	1227 (10.8%)
4+ year	2448 (21.6%)
Experienced intimate partner intimidation and control, *N* (%)	1465 (13.5%)
Experienced harassment by spouse or significant other, *N* (%)	1040 (9.2%)
Experienced harassment by ex-spouse or ex-significant other, *N* (%)	1509 (13.3%)
Experienced harassment by other, *N* (%)	1777 (15.7%)

**Fig. 2:**
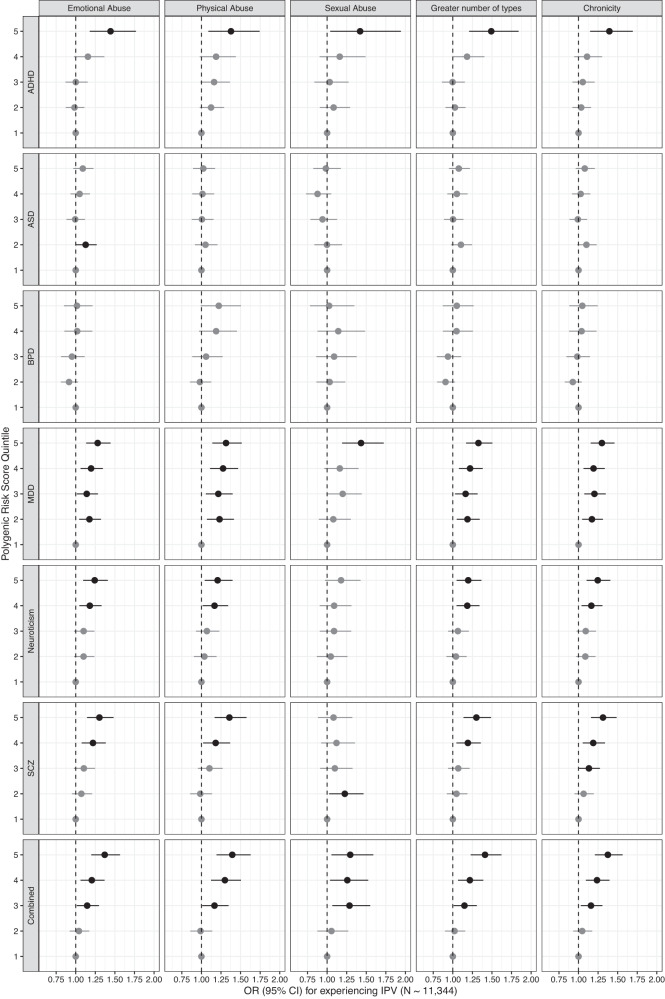
Odds ratios and 95% confidence intervals (CI) associated with quintiles of mental disorder polygenic risk score (PRS) for experiencing emotional, physical, or sexual abuse, greater number of types of IPV, and more chronic IPV, adjusted for genomic assay, and the top 10 principal components of genetic ancestry.

Participants in the highest quintile of PRS for ADHD were at increased risk for experiencing intimate partner emotional (OR = 1.44, 95% CI: 1.18, 1.77, Fig. 2; Supplementary Table 2), physical (OR = 1.38, 95% CI: 1.09, 1.74), and sexual IPV (OR = 1.42, 95% CI: 1.04, 1.94) as well as more types of IPV (OR = 1.49, 95% CI: 1.21, 1.84) and chronic IPV (OR = 1.39, 95% CI: 1.15, 1.69) as compared to the lowest quintile. In tests of trend, increasing ADHD PRS was associated with experience of emotional (OR = 1.12, 95% CI: 1.05, 1.20) and physical IPV (OR = 1.14, 95% CI: 1.05, 1.23), but not sexual IPV (OR = 1.08, 95% CI: 0.98, 1.20; Supplementary Table 2). Participants with PRS in the top quintile for MDD (OR range: 1.28–1.43), neuroticism (OR range: (1.18–1.25), and schizophrenia (OR range: 1.13–1.36) were also at increased risk of experiencing emotional, physical, and sexual IPV (MDD only), as well as more types of IPV and more chronic IPV (Fig. 2; Supplementary Table 2) as compared to the bottom quintile. Tests of trend were significant for polygenic risk for each of these outcome-trait pairs, except for the experience of sexual IPV and the neuroticism and schizophrenia PRS (Supplementary Table 2). The highest quintile of combined PRS for mental illness was associated with experiencing emotional (OR = 1.37, 95% CI: 1.20, 1.57), physical (OR = 1.39, 95% CI: 1.19, 1.63), and sexual IPV (OR = 1.30, 95% CI: 1.06, 1.59), as well as more types of IPV (OR = 1.41, 95% CI: 1.23, 1.62) and more chronic IPV (OR = 1.37, 95% CI: 1.21, 1.56) as compared to those in the lowest quintile. Models including PRS for all six traits, excluding the combined PRS, yielded similar results (see Supplementary Fig. 2).

**Fig. 3:**
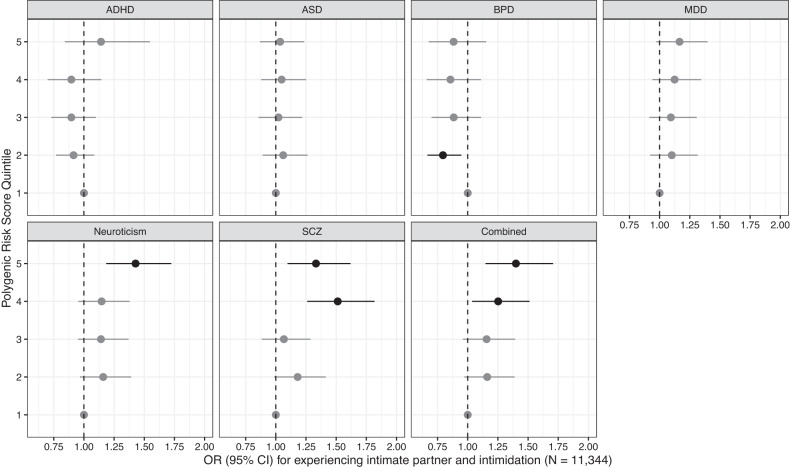
Odds ratios and 95% confidence intervals (CI) associated with quintiles of each mental disorder polygenic risk score (PRS) for experiencing intimate partner intimidation and control as measured by a score ≥20 on the RAT, adjusted for parental education, parental occupation, genomic assay, and the top 10 principal components of genetic ancestry.

Participants in the highest quintile of PRS for neuroticism (OR = 1.43 95% CI: 1.18, 1.72), schizophrenia (OR = 1.33 95% CI: 1.10, 1.62), and the combined PRS (OR = 1.40 95% CI: 1.15, 1.71) were at higher risk (Fig. 3; Supplementary Table 3) for experiencing intimate partner violence and control compared to those in the lowest quintile. Participants in the second quintile of genetic risk for BPD were less likely to experience intimate partner violence and control. However, increasing quintiles of genetic risk were not associated, nor was a test of trend. Tests of the trend for neuroticism, schizophrenia, and the combined PRS were statistically significant (Supplementary Table 3). Associations between each PRS and experience of intimate partner violence and control were consistent when all PRS were included in a single model (see Supplementary Fig. 3).

**Fig. 4:**
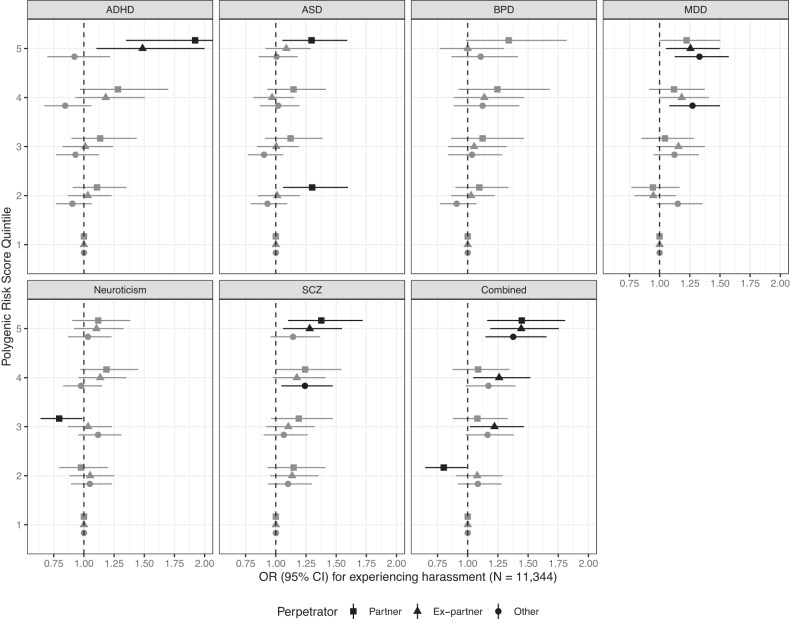
Odds ratios and 95% confidence intervals (CI) associated with quintiles of mental disorder polygenic risk score (PRS) for experiencing harassment from a partner (spouse or significant other), ex-partner (ex-spouse or ex-significant other), or other perpetrator, adjusted for parental education, parental occupation, genomic assay, and the top 10 principal components of genetic ancestry. The association between the ADHD PRS and experiencing harassment by a partner was (OR = 1.91; 95% CI: 1.34, 2.72).

Participants in the highest quintile of PRS for ADHD (OR = 1.92, 95% CI: 1.35, 2.74), ASD (OR = 1.30, 95% CI: 1.06, 1.59), schizophrenia (OR = 1.38, 95% CI: 1.10, 1.72), and overall genetic risk (OR = 1.45, 95% CI: 1.16, 1.81) were likely to experience harassment by a partner (Fig. 4; Supplementary Table 4). Participants in the highest quintile of PRS for ADHD, MDD, schizophrenia, and overall genetic risk were at increased risk of experiencing harassment by an ex-partner (OR range: 1.28–1.49), while participants in the highest quintile of PRS for MDD (OR = 1.33, 95% CI: 1.13, 1.57) and overall PRS (OR = 1.38, 95% CI:1.15, 1.65) were at increased risk for experiencing harassment from another person. Results were consistent when all six PRS were included in a single model for each type of harassment (see Supplementary Fig. 4).

## Discussion

In this study of 11,344 women, PRS for ADHD, MDD, neuroticism, schizophrenia, and combined mental illness were consistently associated with experience of physical, emotional, and sexual IPV, number of types of IPV experienced, and chronicity of IPV. Beyond the experience of IPV, we found that PRS for neuroticism, schizophrenia, and overall genetic risk were also associated with intimate partner intimidation and control. PRS for ADHD, ASD, MDD, schizophrenia, and combined mental illness were significantly associated with harassment from a current or former partner.

Although previous work in this cohort and another have found that genetic risk for BPD was associated with the experience of childhood abuse [51, 52], the BPD PRS was not associated with increased risk for any type of IPV and was mildly protective for the experience of intimate partner intimidation and control. One possible reason for the lack of associations with BPD is that genetic risk for BDP has been shown to be genetically correlated with higher educational attainment [33], which has been shown to be a protective factor for IPV [53–55]. Any increased risk for experiencing IPV stemming from genetic risk for BPD may be reduced by increased educational attainment or other protective factors associated with genetic risk for BPD.

These results build upon prior studies, which have found that depression [17] and ADHD [6] are associated with a higher likelihood of subsequently experiencing IPV. Studies examining neuroticism [56, 57] and ASD [58, 59] as predictors of experiencing interpersonal and intimate partner violence have yielded mixed results. However, these studies did not uniformly adjust for identified predictors of IPV that may have confounded the findings, such as childhood abuse [9, 15], low parental educational attainment [60], and low adulthood SES [12, 61], while our study utilized genetic data unaffected by environmental stressors [62, 63] and not subjected to confounding by these factors or to reverse causation as PRS are set at birth and are not influenced by later experiences of IPV.

Our results expand upon previous findings that genetic risk for mental illness is positively associated with the likelihood of experiencing stressful life events such as childhood abuse [52, 64] and trauma [65, 66]. Multiple pathways potentially link genetic risk for mental illness with risk of IPV victimization, through selection of romantic partners, dynamics within relationships, and ability to leave abusive relationships. First, childhood factors that were not measured in the present study, such as parents modeling healthy interpersonal relationships [67, 68], teaching communication skills [69], and positive parenting [70, 71], may account for some of the associations we found. These factors are positively associated with parents’ mental well-being and, therefore likely to be negatively associated with parents’ PRS for mental illness. The absence of these childhood factors is associated with increased IPV risk in offspring. Second, a higher genetic risk for mental illnesses may be linked to cognitive and behavioral factors that increase the risk of experiencing IPV. For example, higher PRS for ASD [72] and schizophrenia [73] have been associated with greater inability to recognize facial emotions in others, which is a risk factor for experiencing IPV[25–27]. Third, genetic risk for mental illness may be associated with reduced emotional, instrumental, and informational social support from friends and family in adulthood, which may affect women’s risk of IPV and impair their ability to exit an abusive relationship [74–76].

Our study is subject to several limitations. First, the PRS for mental illness typically explains a low proportion of variability when predicting out-of-sample outcomes (Nagelkerke *R*^2^ = 0.01, see Supplementary Table 1.). This may have led to attenuated estimates of true associations between genetic risk and IPV. Second, our study was conducted in a sample of female European ancestry nurses. Our analyses should be replicated in a sample with greater demographic diversity. Moreover, the manner in which high PRS for mental illnesses manifests within this sample of high-functioning individuals may vary from that of the general public.

In providing evidence that genetic risk for mental illness is associated with experience of IPV, our study has potential implications for future treatment and prevention efforts. First, screening for and interventions to reduce the risk of IPV should be considered among women seeking treatment for mental illness, where prevalence rates of IPV are higher [77]. Currently, the broad incorporation of IPV screening tools in mental health services is still largely absent [77], and 60% of mental health providers feel unprepared to properly support survivors of IPV [78]. Second, a better understanding of the salience of the multiple possible pathways linking genetic risk for mental illness with risk for IPV may aid in preventing IPV victimization or re-victimization (e.g., difficulties with peer relations, lower academic attainment, and lower self-esteem have been identified as sequelae of ADHD that may lead to other difficulties) [79, 80]. Without blaming victims, if specific traits, behaviors, or circumstances in persons with genetic loading for various mental illnesses were found, for example, to adversely affect the selection of romantic partners, then addressing these factors might reduce the risk of victimization. Third, although our findings do not have immediate clinical implications, a better understanding of the co-occurrence of IPV and genetic risk for mental illness may lead to more personalized treatments for patients with experiences of IPV. For example, it is hypothesized that depression is a heterogeneous disorder in part due to co-occurrence of, and interaction between, adverse environments and genetic risk factors [28]. Future studies may benefit from examining the extent to which risk for IPV results from interactions of familial, relationship, community, and cultural drivers of IPV with genetic loading for mental illness.

### Supplementary information

Supplemental Material

## Data Availability

All data needed to evaluate the conclusions in the paper are present in the paper and/or the Supplementary Materials. Data from the Nurses Health Study are available through the study’s website: https://nurseshealthstudy.org/researchers.

## References

[1] Smith SG, Basile KC, Gilbert LK, Merrick MT, Patel N, Walling M et al. National intimate partner and sexual violence survey (NISVS): 2010–2012 state report. 2017.

[2] Smith S, Chen J, Basile K, Gilbert L, Merrick M, Patel N et al. The National Intimate Partner and Sexual Violence Survey (NISVS): 2010–2012 State Report, 2017.

[3] Rothman EF, Hathaway J, Stidsen A, de Vries HF (2007). How employment helps female victims of intimate partner violence: a qualitative study. J Occup Health Psychol.

[4] Lagdon S, Armour C, Stringer M (2014). Adult experience of mental health outcomes as a result of intimate partner violence victimisation: a systematic review. Eur J Psychotraumatol.

[5] Golding JM (1999). Intimate partner violence as a risk factor for mental disorders: a meta-analysis. J Fam violence.

[6] Wymbs BT, Dawson AE, Suhr JA, Bunford N, Gidycz CA (2017). ADHD symptoms as risk factors for intimate partner violence perpetration and victimization. J Interpers Violence.

[7] Lehrer JA, Buka S, Gortmaker S, Shrier LA (2006). Depressive symptomatology as a predictor of exposure to intimate partner violence among US female adolescents and young adults. Arch Pediatr Adolesc Med.

[8] Jaffee SR, Price TS (2007). Gene–environment correlations: a review of the evidence and implications for prevention of mental illness. Mol Psychiatry.

[9] Widom CS, Czaja S, Dutton MA (2014). Child abuse and neglect and intimate partner violence victimization and perpetration: a prospective investigation. Child Abus Negl.

[10] Daigneault I, Hébert M, McDuff P (2009). Men’s and women’s childhood sexual abuse and victimization in adult partner relationships: a study of risk factors. Child Abus Negl.

[11] Li S, Zhao F, Yu G (2019). Childhood maltreatment and intimate partner violence victimization: a meta-analysis. Child Abus Negl.

[12] Khalifeh H, Hargreaves J, Howard LM, Birdthistle I (2013). Intimate partner violence and socioeconomic deprivation in England: findings from a national cross-sectional survey. Am J Public Health.

[13] Schiff M, Plotnikova M, Dingle K, Williams GM, Najman J, Clavarino A (2014). Does adolescent’s exposure to parental intimate partner conflict and violence predict psychological distress and substance use in young adulthood? A longitudinal study. Child Abus Negl.

[14] Thulin EJ, Heinze JE, Zimmerman MA (2021). Adolescent adverse childhood experiences and risk of adult intimate partner violence. Am J Prev Med.

[15] Krause-Utz A, Mertens LJ, Renn JB, Lucke P, Wöhlke AZ, van Schie CC (2021). Childhood maltreatment, borderline personality features, and coping as predictors of intimate partner violence. J Interpers Violence.

[16] Baselmans BM, Yengo L, van Rheenen W, Wray NR (2021). Risk in relatives, heritability, SNP-based heritability, and genetic correlations in psychiatric disorders: a review. Biol Psychiatry.

[17] Devries KM, Mak JY, Bacchus LJ, Child JC, Falder G, Petzold M (2013). Intimate partner violence and incident depressive symptoms and suicide attempts: a systematic review of longitudinal studies. PLoS Med.

[18] de Oliveira HN, Machado CJ, Guimarães MDC (2012). Factors associated with self-report of sexual violence against men and women with mental disorders in Brazil. Soc Psychiatry Psychiatr Epidemiol.

[19] Sandberg DA, Valdez CE, Engle JL, Menghrajani E (2019). Attachment anxiety as a risk factor for subsequent intimate partner violence victimization: a 6-month prospective study among college women. J Interpers Violence.

[20] Kuijpers KF, Van Der Knaap LM, Winkel FW (2012). Risk of revictimization of intimate partner violence: the role of attachment, anger and violent behavior of the victim. J Fam Violence.

[21] Papadakaki M, Tzamalouka G, Chatzifotiou S, Chliaoutakis J (2008). Seeking for risk factors of intimate partner violence (IPV) in a Greek National Sample: the role of self-esteem. J Interpers Violence.

[22] Saphire-Bernstein S, Way BM, Kim HS, Sherman DK, Taylor SE (2011). Oxytocin receptor gene (OXTR) is related to psychological resources. Proc Natl Acad Sci.

[23] Cohen S, Schulz MS, Liu SR, Halassa M, Waldinger RJ (2015). Empathic accuracy and aggression in couples: individual and dyadic links. J Marriage Fam.

[24] Miu AC, Homberg JR, Lesch K-P Genes, brain, and emotions: interdisciplinary and translational perspectives. Oxford University Press2019.

[25] Turbett K, Jeffery L, Bell J, Burton J, Palermo R. Autistic traits are associated with less precise perceptual integration of face identity. J. Autism Dev. Disord. 2021;52:2168–179. 10.1007/s10803-021-05111-8 34085152

[26] Waddington F, Franke B, Hartman C, Buitelaar JK, Rommelse N, Mota NR (2021). A polygenic risk score analysis of ASD and ADHD across emotion recognition subtypes. Am J Med Genet Part B: Neuropsychiatr Genet.

[27] Cabras C, Mondo M, Diana A, Sechi C (2020). Relationships between Trait Emotional Intelligence, mood states, and future orientation among female Italian victims of Intimate Partner Violence. Heliyon.

[28] Buch AM, Liston C (2021). Dissecting diagnostic heterogeneity in depression by integrating neuroimaging and genetics. Neuropsychopharmacology.

[29] Meerman JJ, Ter Hark SE, Janzing JG, Coenen MJ (2022). The potential of polygenic risk scores to predict antidepressant treatment response in major depression: a systematic review. J Affect Disord.

[30] Connolly EJ, Hayes BE, Boisvert DL, Cooke EM. Intimate partner victimization and depressive symptoms: approaching causal inference using a longitudinal twin design. *J. Quant. Criminol.* 2022;38:517–35.

[31] Demontis D, Walters RK, Martin J, Mattheisen M, Als TD, Agerbo E (2019). Discovery of the first genome-wide significant risk loci for attention deficit/hyperactivity disorder. Nat Genet.

[32] Grove J, Ripke S, Als TD, Mattheisen M, Walters RK, Won H (2019). Identification of common genetic risk variants for autism spectrum disorder. Nat Genet.

[33] Stahl EA, Breen G, Forstner AJ, McQuillin A, Ripke S, Trubetskoy V (2019). Genome-wide association study identifies 30 loci associated with bipolar disorder. Nat Genet.

[34] Wray NR, Ripke S, Mattheisen M, Trzaskowski M, Byrne EM, Abdellaoui A (2018). Genome-wide association analyses identify 44 risk variants and refine the genetic architecture of major depression. Nat Genet.

[35] Luciano M, Hagenaars SP, Davies G, Hill WD, Clarke T-K, Shirali M (2018). Association analysis in over 329,000 individuals identifies 116 independent variants influencing neuroticism. Nat Genet.

[36] Ripke S, Neale BM, Corvin A, Walters JT, Farh K-H, Holmans PA (2014). Biological insights from 108 schizophrenia-associated genetic loci. Nature.

[37] Tworoger SS, Sluss P, Hankinson SE (2006). Association between plasma prolactin concentrations and risk of breast cancer among predominately premenopausal women. Cancer Res.

[38] Consortium GP (2015). A global reference for human genetic variation. Nature.

[39] Duncan L, Shen H, Gelaye B, Meijsen J, Ressler K, Feldman M (2019). Analysis of polygenic risk score usage and performance in diverse human populations. Nat Commun.

[40] Psychiatric GWAS Consortium Bipolar Disorder Working Group. (2011). Large-scale genome-wide association analysis of bipolar disorder identifies a new susceptibility locus near ODZ4. Nat Genet.

[41] Power RA, Tansey KE, Buttenschon HN, Cohen-Woods S, Bigdeli T, Hall LS (2017). Genome-wide association for major depression through age at onset stratification: major depressive disorder working group of the Psychiatric Genomics Consortium. Biol Psychiatry.

[42] Consortium IS (2009). Common polygenic variation contributes to risk of schizophrenia and bipolar disorder. Nature.

[43] Choi SW, O’Reilly PF (2019). PRSice-2: polygenic risk score software for biobank-scale data. GigaScience.

[44] Smith P, Thornton G, DeVellis R, Earp JA, Coker A (2002). A population-based study of the prevalence and distinctiveness of battering, physical assault, and sexual assault in intimate relationships. Violence Women.

[45] Coker A, Pope BO, Smith P, Sanderson M, Hussey J (2001). Assessment of clinical partner violence screening tools. J Am Med Women’s Assoc (1972).

[46] Smith P, Earp JA, DeVellis R (1995). Measuring battering: development of the Women’s Experience With Battering (WEB) Scale. Women’s Health (Hillsdale, NJ).

[47] Tjaden P, Thoennes N. Prevalence, incidence, and consequences of violence against women: findings from the national violence against women survey. Research in Brief. 1998.

[48] Price AL, Patterson NJ, Plenge RM, Weinblatt ME, Shadick NA, Reich D (2006). Principal components analysis corrects for stratification in genome-wide association studies. Nat Genet.

[49] Brant R. Assessing proportionality in the proportional odds model for ordinal logistic regression. *Biometrics* 1990;46:1171–8. 2085632

[50] Team RC. R: a language and environment for statistical computing. http://www.R-project.org/ 2013.

[51] Park Y-M, Shekhtman T, Kelsoe JR (2020). Interaction between adverse childhood experiences and polygenic risk in patients with bipolar disorder. Transl psychiatry.

[52] Ratanatharathorn A, Koenen KC, Chibnik LB, Weisskopf MG, Rich-Edwards JW, Roberts AL (2021). Polygenic risk for autism, attention-deficit hyperactivity disorder, schizophrenia, major depressive disorder, and neuroticism is associated with the experience of childhood abuse. Mol Psychiatry.

[53] Abramsky T, Watts CH, Garcia-Moreno C, Devries K, Kiss L, Ellsberg M (2011). What factors are associated with recent intimate partner violence? Findings from the WHO multi-country study on women’s health and domestic violence. BMC Public Health.

[54] Rickert VI, Wiemann CM, Harrykissoon SD, Berenson AB, Kolb E (2002). The relationship among demographics, reproductive characteristics, and intimate partner violence. Am J Obstet Gynecol.

[55] Sanz-Barbero B, Barón N, Vives-Cases C (2019). Prevalence, associated factors and health impact of intimate partner violence against women in different life stages. PLoS One.

[56] Ulloa EC, Hammett JF, O’Neal DN, Lydston EE, Leon Aramburo LF. The big five personality traits and intimate partner violence: findings from a large, nationally representative sample. Violence Vict. 2016;31:1100–15. 10.1891/0886-6708.VV-D-15-00055 PMC11294914 27640426

[57] Carton H, Egan V (2017). The dark triad and intimate partner violence. Pers Individ Differ.

[58] Weiss JA, Fardella MA (2018). Victimization and perpetration experiences of adults with autism. Front Psychiatry.

[59] Roberts AL, Koenen KC, Lyall K, Robinson EB, Weisskopf MG (2015). Association of autistic traits in adulthood with childhood abuse, interpersonal victimization, and posttraumatic stress. Child Abus Negl.

[60] Yakubovich AR, Stöckl H, Murray J, Melendez-Torres GJ, Steinert JI, Glavin CEY (2018). Risk and protective factors for intimate partner violence against women: systematic review and meta-analyses of prospective-longitudinal studies. Am J Public Health.

[61] Reichel D (2017). Determinants of intimate partner violence in europe: the role of socioeconomic status, inequality, and partner behavior. J Interpers Violence.

[62] Emdin CA, Khera AV, Kathiresan S (2017). Mendelian randomization. JAMA.

[63] Smith GD, Ebrahim S (2007). Mendelian randomization: genetic variants as instruments for strengthening causal inference in observational studies. BioSoc Surveys.

[64] Dalvie S, Maihofer AX, Coleman JR, Bradley B, Breen G, Brick LA (2020). Genomic influences on self-reported childhood maltreatment. Transl Psychiatry.

[65] Colodro-Conde L, Couvy-Duchesne B, Zhu G, Coventry WL, Byrne EM, Gordon S (2018). A direct test of the diathesis–stress model for depression. Mol Psychiatry.

[66] Amstadter AB, Maihofer A, Koenen KC, Nievergelt C (2021). Molecular genetics of trauma exposure and PTSD. Eur J Psychotraumatol.

[67] Breslau J, Miller E, Jin R, Sampson N, Alonso J, Andrade L (2011). A multinational study of mental disorders, marriage, and divorce. Acta Psychiatr Scand.

[68] Xia M, Fosco GM, Lippold MA, Feinberg ME (2018). A developmental perspective on young adult romantic relationships: Examining family and individual factors in adolescence. J Youth Adolesc.

[69] Vance YH, Huntley SJ, Espie J, Bentall R, Tai S (2008). Parental communication style and family relationships in children of bipolar parents. Br J Clin Psychol.

[70] Shaw DS, Galán CA, Lemery-Chalfant K, Dishion TJ, Elam KK, Wilson MN (2019). Trajectories and predictors of children’s early-starting conduct problems: child, family, genetic, and intervention effects. Dev Psychopathol.

[71] Raby KL, Lawler JM, Shlafer RJ, Hesemeyer PS, Collins WA, Sroufe LA (2015). The interpersonal antecedents of supportive parenting: a prospective, longitudinal study from infancy to adulthood. Dev Psychol.

[72] Wendt FR, Carvalho CM, Pathak GA, Gelernter J, Polimanti R (2020). Polygenic risk for autism spectrum disorder associates with anger recognition in a neurodevelopment-focused phenome-wide scan of unaffected youths from a population-based cohort. PLoS Genet.

[73] Tripoli G, Quattrone D, Ferraro L, Gayer-Anderson C, La Cascia C, Richards A (2022). Facial emotion recognition in psychosis and associations with polygenic risk for schizophrenia. Findings from the multi-centre EU-GEI case-control study. Front Psychiatry.

[74] Reinhard MA, Dewald-Kaufmann J, Wuestenberg T, Musil R, Barton BB, Jobst A (2020). The vicious circle of social exclusion and psychopathology: A systematic review of experimental ostracism research in psychiatric disorders. Eur Arch Psychiatry Clin Neurosci.

[75] Choi AW-M, Wong JY-H, Lo RT-F, Chan P-Y, Wong JK-S, Lau C-L (2018). Intimate partner violence victims’ acceptance and refusal of on-site counseling in emergency departments: predictors of help-seeking behavior explored through a 5-year medical chart review. Prev Med.

[76] Dias NG, Costa D, Soares J, Hatzidimitriadou E, Ioannidi-Kapolou E, Lindert J (2019). Social support and the intimate partner violence victimization among adults from six European countries. Fam Pract.

[77] Arkins B, Begley C, Higgins A (2016). Measures for screening for intimate partner violence: a systematic review. J Psychiatr Ment Health Nurs.

[78] Stewart DE, Chandra PS (2017). WPA international competency-based curriculum for mental health providers on intimate partner violence and sexual violence against women. World Psychiatry.

[79] Niolon PH, Control CfD, Prevention. Preventing intimate partner violence across the lifespan: a technical package of programs, policies, and practices. Government Printing Office, 2017.

[80] Powell V, Riglin L, Hammerton G, Eyre O, Martin J, Anney R (2020). What explains the link between childhood ADHD and adolescent depression? Investigating the role of peer relationships and academic attainment. Eur Child Adolesc Psychiatry.

