# Supplementary material for: Polygenic risk for major depression, attention deficit hyperactivity disorder, neuroticism, and schizophrenia are correlated with experience of intimate partner violence
Source: Transl Psychiatry. 2024 Feb 26;14:119. doi: 10.1038/s41398-024-02814-1 (PMC10897413; doi:10.1038/s41398-024-02814-1)
Supplement: Supplementary file 1 — Supplemental Material [file 41398_2024_2814_MOESM1_ESM.docx]

**Supplemental Table 1.** Genome-wide association study Summary Statistics and parameters for Polygenic Risk Scores (PRS)

| **Disorder** | **Abbreviation** | **Citation** | **PRS p-value threshold** | **Nagelkerke's R2** | **MAF** | **INFO** | **Clumping r2** | **Clumping kb** |
| --- | --- | --- | --- | --- | --- | --- | --- | --- |
| Attention-Deficit/Hyperactivity Disorder | ADHD | Demontis et al. 2019 | 0.1 | 0.0103 | 0.01 | 0.8 | >0.25 | 500 |
| Autism Spectrum Disorder | ASD | Grove et al. 2019 | 0.1 | 0.0245 | 0.05 | 0.9 | >0.1 | 500 |
| Bipolar Disorder | BPD | Stahl et al. 2019 | 0.01 | 0.04 | 0.05 | 0.9 | >0.1 | 500 |
| Major Depressive Disorder | MDD | Wray et al. 2018 | 0.05 | 0.05 | 0.01 | 0.9 | >0.1 | 500 |
| Neuroticism | Neuroticism | Luciano et al. 2018 | 0.05 | 0.0279 | 0.01 | 0.9 | 0.25 | 250 |
| Schizophrenia | SCZ | Ripke et al. 2014 | 0.05 | 0.184 | - | - | - | - |

* MAF = Minor allele frequency; INFO = imputation info score; Clumping r2 = correlation between SNPs with the clumping kb and an index SNP deemed necessary to be considered part of the same locus; Clumping kb = number of kilobase pairs away from an index SNP a variant can be in order to be considered part of the same locus

**Supplemental Table 2.** Odds ratios and 95% confidence intervals for quintiles, trend, and continuous trait PRS on IPV outcomes.

| **Trait** | **Quintile** | **Emotional Abuse** | **Physical Abuse** | **Sexual Abuse** | **Greater number of types** | **Chronicity** |
| --- | --- | --- | --- | --- | --- | --- |
| ADHD | 1 | 1 (Reference) | 1 (Reference) | 1 (Reference) | 1 (Reference) | 1 (Reference) |
| ADHD | 2 | 0.98 (0.87, 1) | 1.12 (0.98, 1) | 1.08 (0.91, 1) | 1.03 (0.91, 1) | 1.03 (0.92, 1) |
| ADHD | 3 | 1 (0.87, 1) | 1.16 (0.99, 1) | 1.03 (0.84, 1) | 1 (0.86, 1) | 1.05 (0.92, 1) |
| ADHD | 4 | 1.16 (0.98, 1) | 1.19 (0.98, 1) | 1.16 (0.9, 1) | 1.18 (0.99, 1) | 1.11 (0.94, 1) |
| ADHD | 5 | 1.44 (1.18, 2) | 1.38 (1.09, 2) | 1.42 (1.04, 2) | 1.49 (1.21, 2) | 1.39 (1.15, 2) |
| ADHD | Trend | 1.08 (1.03, 1) | 1.07 (1.02, 1) | 1.07 (0.99, 1) | 1.08 (1.03, 1) | 1.07 (1.02, 1) |
| ADHD | Continuous | 1.12 (1.05, 1) | 1.14 (1.05, 1) | 1.08 (0.98, 1) | 1.13 (1.05, 1) | 1.11 (1.04, 1) |
| ASD | 1 | 1 (Reference) | 1 (Reference) | 1 (Reference) | 1 (Reference) | 1 (Reference) |
| ASD | 2 | 1.13 (1, 1) | 1.05 (0.92, 1) | 1 (0.84, 1) | 1.1 (0.98, 1) | 1.1 (0.98, 1) |
| ASD | 3 | 0.99 (0.88, 1) | 1.01 (0.88, 1) | 0.94 (0.79, 1) | 1 (0.89, 1) | 0.99 (0.88, 1) |
| ASD | 4 | 1.05 (0.93, 1) | 1.01 (0.88, 1) | 0.88 (0.73, 1) | 1.05 (0.93, 1) | 1.03 (0.92, 1) |
| ASD | 5 | 1.09 (0.97, 1) | 1.02 (0.89, 1) | 0.98 (0.82, 1) | 1.08 (0.95, 1) | 1.08 (0.96, 1) |
| ASD | Trend | 1.01 (0.98, 1) | 1 (0.97, 1) | 0.98 (0.95, 1) | 1.01 (0.98, 1) | 1.01 (0.98, 1) |
| ASD | Continuous | 1.01 (0.98, 1) | 1 (0.96, 1) | 0.98 (0.93, 1) | 1.01 (0.98, 1) | 1.01 (0.98, 1) |
| BPD | 1 | 1 (Reference) | 1 (Reference) | 1 (Reference) | 1 (Reference) | 1 (Reference) |
| BPD | 2 | 0.91 (0.81, 1) | 0.98 (0.85, 1) | 1.03 (0.86, 1) | 0.91 (0.8, 1) | 0.93 (0.83, 1) |
| BPD | 3 | 0.95 (0.81, 1) | 1.06 (0.88, 1) | 1.09 (0.86, 1) | 0.94 (0.8, 1) | 0.98 (0.85, 1) |
| BPD | 4 | 1.02 (0.86, 1) | 1.19 (0.97, 1) | 1.14 (0.88, 1) | 1.05 (0.87, 1) | 1.04 (0.88, 1) |
| BPD | 5 | 1.02 (0.85, 1) | 1.22 (0.99, 2) | 1.03 (0.78, 1) | 1.05 (0.87, 1) | 1.05 (0.88, 1) |
| BPD | Trend | 1.01 (0.97, 1) | 1.06 (1.01, 1) | 1 (0.94, 1) | 1.03 (0.98, 1) | 1.02 (0.98, 1) |
| BPD | Continuous | 1.02 (0.96, 1) | 1.05 (0.98, 1) | 1.01 (0.92, 1) | 1.03 (0.97, 1) | 1.02 (0.96, 1) |
| MDD | 1 | 1 (Reference) | 1 (Reference) | 1 (Reference) | 1 (Reference) | 1 (Reference) |
| MDD | 2 | 1.18 (1.04, 1) | 1.23 (1.07, 1) | 1.08 (0.89, 1) | 1.19 (1.05, 1) | 1.17 (1.05, 1) |
| MDD | 3 | 1.14 (1.01, 1) | 1.22 (1.06, 1) | 1.2 (1, 1) | 1.16 (1.03, 1) | 1.2 (1.07, 1) |
| MDD | 4 | 1.2 (1.06, 1) | 1.28 (1.11, 1) | 1.16 (0.96, 1) | 1.22 (1.08, 1) | 1.19 (1.06, 1) |
| MDD | 5 | 1.28 (1.13, 1) | 1.31 (1.14, 2) | 1.43 (1.19, 2) | 1.33 (1.17, 2) | 1.3 (1.16, 1) |
| MDD | Trend | 1.05 (1.02, 1) | 1.06 (1.03, 1) | 1.08 (1.04, 1) | 1.06 (1.03, 1) | 1.06 (1.03, 1) |
| MDD | Continuous | 1.09 (1.05, 1) | 1.1 (1.05, 1) | 1.14 (1.08, 1) | 1.1 (1.06, 1) | 1.09 (1.05, 1) |
| Neuroticism | 1 | 1 (Reference) | 1 (Reference) | 1 (Reference) | 1 (Reference) | 1 (Reference) |
| Neuroticism | 2 | 1.1 (0.98, 1) | 1.04 (0.9, 1) | 1.05 (0.87, 1) | 1.04 (0.92, 1) | 1.09 (0.97, 1) |
| Neuroticism | 3 | 1.1 (0.98, 1) | 1.07 (0.93, 1) | 1.09 (0.91, 1) | 1.06 (0.94, 1) | 1.09 (0.97, 1) |
| Neuroticism | 4 | 1.18 (1.05, 1) | 1.17 (1.02, 1) | 1.09 (0.91, 1) | 1.18 (1.05, 1) | 1.16 (1.04, 1) |
| Neuroticism | 5 | 1.24 (1.09, 1) | 1.21 (1.04, 1) | 1.18 (0.97, 1) | 1.2 (1.05, 1) | 1.25 (1.1, 1) |
| Neuroticism | Trend | 1.05 (1.02, 1) | 1.05 (1.02, 1) | 1.04 (0.99, 1) | 1.05 (1.02, 1) | 1.05 (1.02, 1) |
| Neuroticism | Continuous | 1.07 (1.02, 1) | 1.07 (1.03, 1) | 1.07 (1.01, 1) | 1.07 (1.03, 1) | 1.07 (1.03, 1) |
| SCZ | 1 | 1 (Reference) | 1 (Reference) | 1 (Reference) | 1 (Reference) | 1 (Reference) |
| SCZ | 2 | 1.07 (0.95, 1) | 0.99 (0.86, 1) | 1.23 (1.03, 1) | 1.05 (0.92, 1) | 1.06 (0.95, 1) |
| SCZ | 3 | 1.1 (0.98, 1) | 1.1 (0.96, 1) | 1.1 (0.91, 1) | 1.07 (0.94, 1) | 1.13 (1.01, 1) |
| SCZ | 4 | 1.22 (1.08, 1) | 1.18 (1.02, 1) | 1.12 (0.92, 1) | 1.19 (1.05, 1) | 1.19 (1.05, 1) |
| SCZ | 5 | 1.3 (1.14, 1) | 1.36 (1.17, 2) | 1.08 (0.88, 1) | 1.3 (1.14, 1) | 1.31 (1.16, 1) |
| SCZ | Trend | 1.07 (1.04, 1) | 1.08 (1.05, 1) | 1.01 (0.96, 1) | 1.07 (1.04, 1) | 1.07 (1.04, 1) |
| SCZ | Continuous | 1.09 (1.04, 1) | 1.11 (1.06, 1) | 1.03 (0.96, 1) | 1.09 (1.04, 1) | 1.08 (1.04, 1) |
| Combined | 1 | 1 (Reference) | 1 (Reference) | 1 (Reference) | 1 (Reference) | 1 (Reference) |
| Combined | 2 | 1.04 (0.92, 1) | 0.99 (0.86, 1) | 1.06 (0.88, 1) | 1.02 (0.9, 1) | 1.05 (0.93, 1) |
| Combined | 3 | 1.15 (1.01, 1) | 1.17 (1.01, 1) | 1.29 (1.07, 2) | 1.15 (1.01, 1) | 1.16 (1.03, 1) |
| Combined | 4 | 1.21 (1.06, 1) | 1.3 (1.12, 2) | 1.26 (1.04, 2) | 1.22 (1.07, 1) | 1.24 (1.09, 1) |
| Combined | 5 | 1.37 (1.2, 2) | 1.39 (1.19, 2) | 1.3 (1.06, 2) | 1.41 (1.23, 2) | 1.37 (1.21, 2) |
| Combined | Trend | 1.08 (1.05, 1) | 1.1 (1.06, 1) | 1.07 (1.02, 1) | 1.09 (1.06, 1) | 1.08 (1.05, 1) |
| Combined | Continuous | 1.13 (1.08, 1) | 1.15 (1.09, 1) | 1.11 (1.04, 1) | 1.14 (1.09, 1) | 1.13 (1.08, 1) |

**Supplemental Table 3.** Odds ratios and 95% confidence intervals for quintiles, trend, and continuous trait PRS on Intimate partner intimidation and control.

| **Trait** | **Quintile** | **OR (95% CI)** |
| --- | --- | --- |
| ADHD | 1 | 1 (Reference) |
| ADHD | 2 | 0.91 (0.77, 1.09) |
| ADHD | 3 | 0.9 (0.73, 1.1) |
| ADHD | 4 | 0.9 (0.7, 1.14) |
| ADHD | 5 | 1.14 (0.84, 1.55) |
| ADHD | Trend | 1 (0.93, 1.08) |
| ADHD | Continuous | 0.99 (0.9, 1.1) |
| ASD | 1 | 1 (Reference) |
| ASD | 2 | 1.06 (0.89, 1.26) |
| ASD | 3 | 1.02 (0.86, 1.22) |
| ASD | 4 | 1.05 (0.88, 1.25) |
| ASD | 5 | 1.04 (0.87, 1.23) |
| ASD | Trend | 1.01 (0.97, 1.05) |
| ASD | Continuous | 1.03 (0.97, 1.08) |
| BPD | 1 | 1 (Reference) |
| BPD | 2 | 0.79 (0.67, 0.95) |
| BPD | 3 | 0.88 (0.7, 1.11) |
| BPD | 4 | 0.86 (0.66, 1.11) |
| BPD | 5 | 0.88 (0.68, 1.15) |
| BPD | Trend | 0.98 (0.92, 1.04) |
| BPD | Continuous | 0.97 (0.88, 1.06) |
| MDD | 1 | 1 (Reference) |
| MDD | 2 | 1.1 (0.92, 1.32) |
| MDD | 3 | 1.09 (0.91, 1.31) |
| MDD | 4 | 1.12 (0.94, 1.34) |
| MDD | 5 | 1.16 (0.97, 1.4) |
| MDD | Trend | 1.03 (0.99, 1.08) |
| MDD | Continuous | 1.06 (1, 1.12) |
| Neuroticism | 1 | 1 (Reference) |
| Neuroticism | 2 | 1.16 (0.97, 1.39) |
| Neuroticism | 3 | 1.14 (0.95, 1.37) |
| Neuroticism | 4 | 1.15 (0.95, 1.38) |
| Neuroticism | 5 | 1.43 (1.18, 1.72) |
| Neuroticism | Trend | 1.07 (1.03, 1.12) |
| Neuroticism | Continuous | 1.1 (1.04, 1.17) |
| SCZ | 1 | 1 (Reference) |
| SCZ | 2 | 1.18 (0.99, 1.41) |
| SCZ | 3 | 1.07 (0.88, 1.29) |
| SCZ | 4 | 1.51 (1.26, 1.82) |
| SCZ | 5 | 1.33 (1.1, 1.62) |
| SCZ | Trend | 1.09 (1.04, 1.13) |
| SCZ | Continuous | 1.14 (1.07, 1.21) |
| Combined | 1 | 1 (Reference) |
| Combined | 2 | 1.16 (0.97, 1.39) |
| Combined | 3 | 1.16 (0.96, 1.39) |
| Combined | 4 | 1.25 (1.04, 1.51) |
| Combined | 5 | 1.4 (1.15, 1.71) |
| Combined | Trend | 1.08 (1.03, 1.13) |
| Combined | Continuous | 1.13 (1.06, 1.2) |

**Supplemental Table 4.** Odds ratios and 95% confidence intervals for quintiles, trend, and continuous trait PRS on harassment by a partner, ex-partner, or other person.

| **Trait** | **quintile** | **Partner** | **Ex-partner** | **Other** |
| --- | --- | --- | --- | --- |
| ADHD | 1 | 1 (Reference) | 1 (Reference) | 1 (Reference) |
| ADHD | 2 | 1.11 (0.91, 1.35) | 1.03 (0.87, 1.23) | 0.9 (0.77, 1.07) |
| ADHD | 3 | 1.13 (0.9, 1.44) | 1.01 (0.82, 1.24) | 0.93 (0.77, 1.13) |
| ADHD | 4 | 1.28 (0.97, 1.7) | 1.18 (0.93, 1.5) | 0.84 (0.67, 1.06) |
| ADHD | 5 | 1.92 (1.35, 2.74) | 1.49 (1.1, 2) | 0.92 (0.7, 1.22) |
| ADHD | Trend | 1.13 (1.04, 1.23) | 1.08 (1.01, 1.16) | 0.97 (0.91, 1.04) |
| ADHD | Continuous | 1.21 (1.08, 1.36) | 1.13 (1.02, 1.25) | 1.01 (0.92, 1.11) |
| ASD | 1 | 1 (Reference) | 1 (Reference) | 1 (Reference) |
| ASD | 2 | 1.3 (1.06, 1.6) | 1.01 (0.85, 1.2) | 0.93 (0.79, 1.09) |
| ASD | 3 | 1.12 (0.91, 1.39) | 1 (0.84, 1.19) | 0.9 (0.77, 1.06) |
| ASD | 4 | 1.15 (0.93, 1.42) | 0.97 (0.81, 1.15) | 1.02 (0.87, 1.2) |
| ASD | 5 | 1.3 (1.06, 1.59) | 1.09 (0.92, 1.29) | 1.01 (0.86, 1.18) |
| ASD | Trend | 1.04 (0.99, 1.09) | 1.01 (0.97, 1.05) | 1.01 (0.97, 1.05) |
| ASD | Continuous | 1.05 (0.99, 1.12) | 1.02 (0.97, 1.08) | 1.02 (0.97, 1.07) |
| BPD | 1 | 1 (Reference) | 1 (Reference) | 1 (Reference) |
| BPD | 2 | 1.1 (0.9, 1.34) | 1.03 (0.86, 1.22) | 0.91 (0.77, 1.07) |
| BPD | 3 | 1.12 (0.86, 1.46) | 1.05 (0.84, 1.32) | 1.04 (0.83, 1.28) |
| BPD | 4 | 1.25 (0.92, 1.68) | 1.14 (0.88, 1.47) | 1.12 (0.88, 1.43) |
| BPD | 5 | 1.34 (0.99, 1.82) | 1 (0.77, 1.3) | 1.11 (0.87, 1.42) |
| BPD | Trend | 1.08 (1, 1.16) | 1 (0.94, 1.06) | 1.03 (0.97, 1.1) |
| BPD | Continuous | 1.09 (0.98, 1.21) | 0.99 (0.91, 1.09) | 1.08 (0.99, 1.17) |
| MDD | 1 | 1 (Reference) | 1 (Reference) | 1 (Reference) |
| MDD | 2 | 0.95 (0.77, 1.17) | 0.95 (0.79, 1.13) | 1.15 (0.98, 1.36) |
| MDD | 3 | 1.04 (0.85, 1.28) | 1.16 (0.97, 1.38) | 1.12 (0.95, 1.33) |
| MDD | 4 | 1.12 (0.91, 1.37) | 1.18 (0.99, 1.41) | 1.27 (1.08, 1.5) |
| MDD | 5 | 1.22 (1, 1.5) | 1.26 (1.05, 1.5) | 1.33 (1.13, 1.57) |
| MDD | Trend | 1.06 (1.01, 1.11) | 1.07 (1.03, 1.11) | 1.07 (1.03, 1.11) |
| MDD | Continuous | 1.09 (1.02, 1.17) | 1.13 (1.07, 1.2) | 1.12 (1.06, 1.18) |
| Neuroticism | 1 | 1 (Reference) | 1 (Reference) | 1 (Reference) |
| Neuroticism | 2 | 0.98 (0.79, 1.2) | 1.05 (0.88, 1.25) | 1.05 (0.89, 1.23) |
| Neuroticism | 3 | 0.79 (0.64, 0.99) | 1.03 (0.87, 1.23) | 1.12 (0.95, 1.31) |
| Neuroticism | 4 | 1.19 (0.97, 1.45) | 1.14 (0.95, 1.35) | 0.98 (0.83, 1.15) |
| Neuroticism | 5 | 1.12 (0.9, 1.38) | 1.1 (0.92, 1.33) | 1.03 (0.87, 1.23) |
| Neuroticism | Trend | 1.04 (0.99, 1.1) | 1.03 (0.99, 1.07) | 1 (0.96, 1.04) |
| Neuroticism | Continuous | 1.06 (0.99, 1.13) | 1.04 (0.99, 1.11) | 1.02 (0.97, 1.08) |
| SCZ | 1 | 1 (Reference) | 1 (Reference) | 1 (Reference) |
| SCZ | 2 | 1.15 (0.93, 1.41) | 1.13 (0.95, 1.35) | 1.1 (0.93, 1.3) |
| SCZ | 3 | 1.19 (0.96, 1.47) | 1.1 (0.92, 1.32) | 1.07 (0.9, 1.26) |
| SCZ | 4 | 1.24 (1, 1.54) | 1.17 (0.98, 1.41) | 1.24 (1.05, 1.47) |
| SCZ | 5 | 1.38 (1.1, 1.72) | 1.28 (1.06, 1.55) | 1.14 (0.96, 1.37) |
| SCZ | Trend | 1.07 (1.02, 1.13) | 1.05 (1.01, 1.1) | 1.04 (1, 1.08) |
| SCZ | Continuous | 1.1 (1.02, 1.18) | 1.08 (1.01, 1.14) | 1.07 (1.01, 1.14) |
| Combined | 1 | 1 (Reference) | 1 (Reference) | 1 (Reference) |
| Combined | 2 | 0.8 (0.65, 0.99) | 1.08 (0.9, 1.29) | 1.08 (0.92, 1.28) |
| Combined | 3 | 1.08 (0.88, 1.33) | 1.22 (1.02, 1.47) | 1.17 (0.98, 1.38) |
| Combined | 4 | 1.09 (0.88, 1.35) | 1.26 (1.05, 1.52) | 1.17 (0.98, 1.4) |
| Combined | 5 | 1.45 (1.16, 1.81) | 1.44 (1.19, 1.75) | 1.38 (1.15, 1.65) |
| Combined | Trend | 1.11 (1.05, 1.17) | 1.09 (1.05, 1.14) | 1.07 (1.03, 1.12) |
| Combined | Continuous | 1.18 (1.09, 1.27) | 1.13 (1.06, 1.21) | 1.11 (1.05, 1.18) |

**Supplemental Figure 1. Correlations between PRS for ADHD, ASD, BPD, MDD, neuroticism, and MDD.**

**
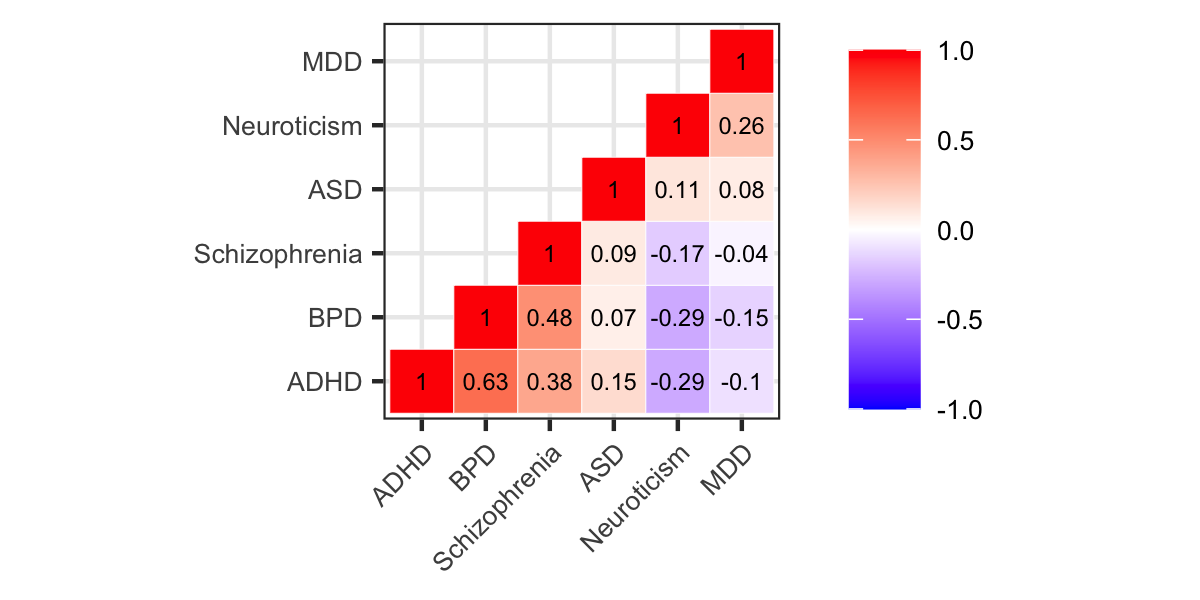
**

**Supplemental Figure 2. Odds ratios and 95% confidence intervals (CI) associated with quintiles of mental disorder polygenic risk score (PRS) for experiencing emotional, physical, or sexual abuse, greater number of types of IPV, and more chronic IPV, adjusted for genomic assay, and the top 10 principal components of genetic ancestry. In blue is the original analysis where only 1 PRS is included in the model at a time and in red is the mutually adjusted model where all 6 PRS are included at once.**

**Supplemental Figure 3. Odds ratios and 95% confidence intervals (CI) associated with quintiles of each mental disorder polygenic risk score (PRS) for experiencing intimate partner intimidation and control as measured by a score ≥20 on the RAT, adjusted for parental education, parental occupation, genomic assay, and the top 10 principal components of genetic ancestry. In blue is the original analysis where only 1 PRS is included in the model at a time and in red is the mutually adjusted model where all 6 PRS are included at once.**

**Supplemental Figure 4. Odds ratios and 95% confidence intervals (CI) associated with quintiles of mental disorder polygenic risk score (PRS) for experiencing harassment from a partner (spouse or significant other), ex-partner (ex-spouse or ex-significant other), or other perpetrator. In blue is the original analysis where only 1 PRS is included in the model at a time and in red is the mutually adjusted model where all 6 PRS are included at once.**
